# Traumatic Brain Injury and Alzheimer’s Disease: A Shared Neurovascular Hypothesis

**DOI:** 10.1177/26331055251323292

**Published:** 2025-03-20

**Authors:** Gabrielle Cognacq, Jonathan E Attwood, Gabriele C DeLuca

**Affiliations:** 1 John Radcliffe Hospital, University of Oxford, Headley Way, Oxford, Oxfordshire, UK; 2 Nuffield Department of Clinical Neurosciences, Level 6 West Wing, John Radcliffe Hospital, Headley Way, Oxford, Oxfordshire, UK

**Keywords:** Alzheimer’s disease, traumatic brain injury, neurovascular unit, blood-brain barrier, fibrinogen

## Abstract

Traumatic brain injury (TBI) is a modifiable risk factor for Alzheimer’s disease (AD). TBI and AD share several histopathological hallmarks: namely, beta-amyloid aggregation, tau hyperphosphorylation, and plasma protein infiltration. The relative contributions of these proteinopathies and their interplay in the pathogenesis of both conditions remains unclear although important differences are emerging. This review synthesises emerging evidence for the critical role of the neurovascular unit in mediating protein accumulation and neurotoxicity in both TBI and AD. We propose a shared pathogenic cascade centred on a neurovascular unit, in which increased blood-brain barrier permeability induces a series of noxious mechanisms leading to neuronal loss, synaptic dysfunction and ultimately cognitive dysfunction in both conditions. We explore the application of this hypothesis to outstanding research questions and potential treatments for TBI and AD, as well as other neurodegenerative and neuroinflammatory conditions. Limitations of this hypothesis, including the challenges of establishing a causal relationship between neurovascular damage and proteinopathies, are also discussed.

## Introduction

Traumatic brain injury (TBI) occurs when an external force exerted on the brain results in impaired brain function, or other evidence of brain pathology.^1,2^ TBI encompasses a wide range of conditions, from sports-related concussions to brain injuries caused by falls or road traffic collisions to penetrating injuries sustained in the context of combat or other major trauma. It is increasingly recognised that TBI in any form can trigger pathophysiological processes which, in some cases, persist long after the initial insult.^3,4^ Sustaining repeated subconcussive head impacts is also associated with long-term neuropathological changes.^5,6^

It has been shown that individuals who sustain TBI are at increased risk of developing a range of neurological and psychiatric conditions later in life, including dementia,^7-9^ ischaemic stroke,^10^ epilepsy,^11^ depression,^12^ and sleep disorders.^13^ Indeed, TBI is now widely recognised as a modifiable risk factor for dementia,^9^ with the strongest evidence supporting a link between moderate-severe TBI and Alzheimer’s disease (AD) in an age-dependent manner.^7,14^ AD is the most common form of dementia and is characterised by neuronal loss, synaptic dysfunction and progressive cognitive decline.^15-18^ TBI and AD share similar vascular systemic risk factors (Table 1) and characteristic histopathological features which include the formation of senile beta-amyloid (Aβ) plaques and tau aggregates in the brain parenchyma as well as disruption of the neurovasculature.^19,20^

**Table 1.** Vascular systemic and environmental risk factors that are associated with AD and contribute to post-TBI outcomes.

Risk factors	Alzheimer’s disease	Traumatic brain injury
Ageing	Strongest epigenetic factor for LOAD, associated with BBB alterations particularly in the hippocampus^21^	Exacerbates early BBB opening and dysfunction in TBI^22^
Cardiovascular disease (CVD)	Increases the risk of AD; AD-related amyloidosis is also a risk factor for CVD^23^	Associated with poor post-TBI cognitive outcome^24^
Hypertension	Mediates BBB alterations, exacerbates AD^25^	Associated with poor post-TBI cognitive outcome^24^
Hyperlipidaemia/hypercholesterolaemia	Increases risk of AD^26^	Saturated fat diet associated with altered BBB repair and poor TBI outcome^27^
Atherosclerosis	Increases risk of AD^28^; associated with increased phosphorylation of tau epitopes commonly found in AD^29^	(Effect on TBI outcome undocumented in the literature)
Diabetes mellitus	Increases risk of AD^30^; insulin resistance associated with amyloidosis and oxidative stress^31^; tau and amyloid proteinopathies seen in pancreatic tissues of type 2 diabetes patients^32^	Exacerbates TBI-induced cortical damage and is associated with increased mortality^33^
Alcoholism	Increases risk of AD^30^; increases BBB permeability, Aβ aggregation and tau hyperphosphorylation in CA1 hippocampus^34^	Associated with higher mortality rate^35^
Smoking	Risk factor for AD and vascular dementia^36^; associated with tau hyperphosphorylation^37^	Affects TBI recovery; associated with poor cognitive outcome^36^
Hypoxia/ischaemia	Poor brain perfusion is associated with increased risk of AD and cognitive impairment^38^; early and delayed upregulation of tau-encoding gene expression in the infarcted region following cerebral ischemia^39^; HIF-1α-mediated BACE1 upregulation^40^	(Effect of previous hypoxic/ischaemic events on TBI outcome undocumented in the literature)

The neurovascular unit (NVU) is a dynamic system of interacting cells that are responsible for adequate brain functioning. It is composed of neurons, vascular endothelial and smooth muscle cells, pericytes and glial cells including astrocytes and microglia. The NVU maintains the blood-brain barrier (BBB), which regulates the exchange of cells between the blood and the central nervous system (CNS) by controlling vascular permeability. Under physiological conditions, the BBB prevents entry of plasma-derived products and tightly controls the CNS microenvironment stability to maintain brain function.

In this review, we synthesise emerging evidence that indicates a critical role for NVU disruption and BBB dysfunction in the pathogenesis of both TBI and AD. We propose that a common neurovascular cascade unites these two pathologies and discuss the implications of this for future research and the treatment of both conditions.

## AD and TBI Share Core Pathological Features

### AD pathogenesis

The histopathological hallmarks of AD are extracellular Aβ plaques, resulting from pathological cleavage of the amyloid precursor protein (APP) by secretases, and intracellular neurofibrillary tangles (NFTs) of hyperphosphorylated tau (p-tau), a microtubule-associated protein.^41,42^ These protein aggregates are able to induce perivascular microglial activation and trigger a chronic inflammatory response which can lead to neuronal death, synaptic loss and, in turn, cognitive dysfunction.^43,44^

The initial prevailing theory of AD pathogenesis was the vascular dysregulation hypothesis.^30^ Bradley et al were among the first to compare Braak staging of post-mortem tissues with single photon emission tomography (SPET) scans obtained at multiple timepoints before death: they identified patterns of gradual cerebral perfusion changes which correlated with disease progression.^45^ Consistent with this theory, reduced cerebral blood flow and vascular reactivity along with reduced microvascular density have been shown to be the earliest signs of AD, preceding both tau hyperphosphorylation and tau-induced neuronal loss.^46^ More recently, initial microvascular hypoperfusion and deteriorating capillary integrity and function were shown to correlate with the progressive AD-associated cognitive decline.^47^

However, to date, the most influential theory of AD pathogenesis has been the amyloid cascade hypothesis. According to this theory, Aβ aggregation triggers a variety of downstream effects including p-tau aggregation and neuronal death. This theory grew out of the emergence of genetic studies which identified highly penetrant genes involved in familial AD (fAD) and their role in β-amyloidosis (eg, *APP, PSEN1, PSEN2*). Amyloid deposition in the brain parenchyma and vasculature, including meningeal blood vessels, is recognised as an early pathological feature of AD. Aberrant cleavage of APP results in various conformational aggregates, including soluble monomers and oligomers which readily elongate into protofibrils that in turn serve as growth substrates for mature fibrils, the precursors to Aβ plaques.^18,48,49^ However, more recent findings have challenged the dominance of this hypothesis. It is now understood that the genetic aberrations responsible for fAD do not account for the sporadic late-onset form of AD (LOAD), which represents >95% of AD cases.^50^ Importantly, the extent of Aβ plaques does not correlate with the degree of cognitive decline and neurodegeneration in individuals with AD.^51^ Perhaps most significantly, clinical trials of anti-Aβ immunotherapy have shown that clearing extra-cellular Aβ plaques results in only modest changes to cognition, at best.^52^ However, growing evidence suggests that soluble Aβ oligomers, may play a more direct role in mediating neurotoxicity than Aβ plaques in AD.^53-55^ Lecanemab, a humanised monoclonal antibody approved by the FDA for AD treatment, targets soluble Aβ oligomers and protofibrils.^56-58^ However, the risks of amyloid-related imaging abnormalities and cerebral haemorrhage associated with the treatment were considered by the EMA to outweigh its small reported benefit on cognition, resulting in the refusal of marketing authorisation.^59^

Uncertainty regarding the amyloid cascade hypothesis has coincided with renewed interest in tau pathophysiology.^60^ The spatiotemporal pattern of p-tau pathology in AD is highly conserved and reflects the progression of cognitive symptoms seen in the condition.^61^ NFTs initially form in the entorhinal cortex and hippocampus, structures involved in memory processing, before appearing in anatomically connected areas, including the limbic system and association cortices.^41,62^ This pattern is consistent with the selective neuronal vulnerability (SNV) of CA1 hippocampal neurons, which are specifically prone to degeneration in AD.^63^ The balance of evidence now appears to support a model of AD pathogenesis in which p-tau self-propagates to instigate neurodegeneration in a prion-like fashion.^46,64^ This model is consistent with p-tau mediating Aβ toxicity, although it is notable that soluble tau oligomers can induce neuronal toxicity in the absence of both NFTs and Aβ plaques.^65^ Interestingly, primary age-related tauopathy (PART), which is characterised by the presence of AD-like NFTs in the medial temporal lobe, remains distinct from AD in that it harbours mild, if any, Aβ plaques, and is often not associated with cognitive decline.^66,67^ Notwithstanding the strengths of this model, the factors underlying the formation and spread of pathological p-tau remain unclear.

In this context, a unified theory of AD pathogenesis must account for neurovascular dysregulation as well as the contributions of Aβ and p-tau.

### TBI pathophysiology

The pathophysiology of TBI can be divided into acute and chronic phases. In the acute phase, primary injury results from mechanical forces subjected to the brain and skull, which can lead to both focal and diffuse damage including haemorrhage, haematoma, oedema, and diffuse axonal injury (DAI).^68,69^ DAI is the defining neuropathological feature of TBI. It is present in both mild and severe concussive injuries, in both the acute and chronic post-TBI phases, and is positively correlated to the degree of cognitive decline following TBI.^70^ In the chronic phase of TBI pathophysiology, the initial injury triggers secondary damage through a series of mechanisms including chronic inflammation, ischaemia and neuronal death. Specifically, a combination of increased microglial activation, persistent astrogliosis, macrophage infiltration, and oxidative stress cause white matter damage and network dysfunctions which can persist for several decades after injury.^68,71-73^ Importantly, sustaining TBI at an older age is associated with an increased risk of developing dementia.^14^ Mechanism of injury is another important modulating factor: motor-vehicle accident-related injuries commonly cause DAIs in young adults, whereas falls usually result in mass lesions such as haemorrhages.^74^

There are several parallels between TBI and AD pathophysiology. Altered cerebrovascular autoregulation and reduced cerebral blood flow are key features of the acute phase after TBI.^22^ It is now also clear that both p-tau aggregates and Aβ plaques are features of TBI pathophysiology.^75,76^ Recent studies have demonstrated that TBI can induce altered β-cleavage of APP and tau processing by upregulating the expression of asparaginyl endopeptidase in the acute and subacute post-injury periods, leading to the formation of AD-like intraneuronal and perivascular p-tau NFTs and extracellular Aβ plaques in both mouse models and human brain tissues.^42,77^ Although AD-like SNV of CA1 hippocampal neurons has also been identified in TBI, a wider distribution of p-tau and amyloid aggregates (spreading across extensive cortical areas including white matter) has been observed in TBI, which differs from classical AD pathology.^78-80^ It is also important to note that the emergence of AD-like pathology in the chronic phase of TBI appears to be a distinct entity to the development of chronic traumatic encephalopathy (CTE) following exposure to repetitive head impacts (RHI), although shared mechanisms are likely to be involved.^6^ However, the overall prevalence and clinical relevance of AD-like pathology following TBI remains unclear. For example, recent positron emission tomography (PET) studies have found no significant difference in Aβ and tau tracer levels between TBI patients and healthy controls, despite worse cognitive outcomes in the TBI groups.^81,82^ This suggests the presence of alternative pathological mechanisms by which TBI can produce cognitive dysfunction.

## Evidence of Neurovascular Pathology in Both AD and TBI

### NVU damage and BBB disruption

NVU damage and loss of BBB integrity are well-recognised pathological features of AD that correlate with disease progression.^83^ Microbleeds can be detected in brain MR imaging of patients with LOAD and colocalise with Aβ plaques and infiltration of blood-derived products.^84,85^ Importantly, there is evidence to suggest that BBB and disruption precede Aβ and p-tau pathology. In a mouse model of AD, an increase in BBB permeability was recorded prior to senile plaque formation, and subsequent Aβ aggregation was associated with further degradation of BBB integrity.^86^ In humans, capillary damage and pericyte injury in hippocampal regions have been identified as the earliest signs of cognitive dysfunction among AD patients.^87^ Notably, in this study, early hippocampal BBB breakdown was independent of Aβ and p-tau burden. More recently, an assessment of NVU changes in post-mortem hippocampal and cortical tissues of AD patients revealed a progressive decrease in pericyte coverage which correlated with the Braak stages of AD severity and both Aβ and p-tau pathology.^88^ Although the evidence is not sufficient to support a causal relationship, the data suggest that loss of pericytes might precede and mediate accumulation of Aβ and p-tau. Consistent with this, astrocyte malfunction results in impaired clearance of protein aggregates.^89^ In a more recent study, Kim et al demonstrated that impaired astrocytic autophagy leads to increased levels of Aβ aggregates and p-tau filaments, which correlate with poorer cognitive function in AD mouse models.^90^ Conversely, overexpression of autophagy-related genes in astrocytes is associated with a reduction in Aβ burden and improved behavioural symptoms in vivo,^90^ further highlighting the role of astrocytes in protein clearance. Finally, the deterioration of endothelial glycocalyx (GCX), a nanostructure lining vascular endothelial cell that maintains physiological endothelial function and vessel permeability, is increasingly recognised as a potential contributor to BBB disruption in AD.^91^ Loss of GCX has been demonstrated in AD brain tissue, as well as in vascular dementia and other neurodegenerative diseases, and is associated with elevated neuroinflammation and oxidative stress.^92,93^ While the conflation of evidence supports a role for NVU dysfunction in AD, the mechanisms underlying such NVU changes remain unclear.

Similarly, NVU damage and BBB breakdown are neuropathological features of TBI that correlate with disease progression. Microbleeds are detectable in both the acute and chronic stages after injury and are positively associated with damage severity.^72^ Loss of BBB integrity is recognised as an early sign of cognitive impairment following TBI; several studies have reported BBB breach within minutes and hours after injury in animal models and human brain tissue samples, respectively.^22,94,95^ A number of NVU components appear to be altered following TBI and may contribute to increased BBB permeability. In juvenile rat models of TBI, long-term endothelial cell remodelling and changes in basement membrane phenotypes led to a poor clearance of protein aggregates.^76^ Expression of tight junction-associated proteins was also shown to be downregulated by severe TBI, causing infiltration of blood-borne proteins such as albumin.^96,97^ Moreover, evidence suggests that significant pericyte loss is a frequent acute post-TBI mechanism that can trigger further NVU deterioration.^98^ In a mouse model of TBI, epigenetic changes were associated with downregulated basement membrane proteins resulting from acute pericyte loss, and a reduction in extracellular matrix proteins connecting pericytes to endothelial cells was also observed.^99^ Gonzalez Rodriguez et al also demonstrated significant shedding of endothelial GCX in TBI patients, which was associated with higher odds of complications and mortality.^100^

The mechanisms underlying TBI-induced NVU changes remain unclear. Oxidative stress most likely plays a role, and there is emerging evidence to suggest that increased iron accumulation in the brain is a key source of reactive oxygen species (ROS) in both TBI and AD.^101^ Both haem iron and non-haem iron have been shown to accumulate in the injured brain. The source of haem iron is understood to be the egress of blood from damaged vessels, either as frank haemorrhage in severe TBI or microhaemorrhages detectable with susceptibility-weight imaging (SWI) in concussion.^102^ Non-haem iron (ie, free iron and iron bound to non-haem proteins such as ferritin and transferrin) is thought to be released from degenerating neurons and has been shown to be elevated after a single TBI in mice.^103^ ROS reportedly reduces tight junction protein levels and activates matrix metalloproteinases and aquaporin channels, thereby contributing to the loss of BBB integrity and the formation of vasogenic oedema in TBI.^68,104^ Subsequently, chronic neuroinflammatory processes may mediate long-term BBB disruption which can persist for years after the initial injury, and which is closely associated with cognitive outcome. Interestingly, in a mouse model of TBI, injection of the mast cell inhibitor drug cromolyn reverses TBI-induced pericyte and tight junction loss, resorbs oedema, and rescues cognitive function.^95,104^

### Shared genetic, systemic, and environmental risk factors

A series of vascular risk factors have been associated with AD, further supporting the role of NVU disruption and BBB dysfunction in the pathogenesis of the condition. The strongest genetic risk factor for LOAD is the Apolipoprotein E4 allele (APOE4). APOE4 is recognised to induce pericyte degeneration and BBB breakdown by interfering with TREM2 signalling, which plays a role in microglial dysfunction, and various other proinflammatory signalling pathways.^105-108^ In a recent study, BBB breakdown was investigated in APOE4 carriers who were either cognitively normal or at the earliest symptomatic stage of AD.^105^ This showed that BBB breakdown in hippocampal and parahippocampal regions was greater in APOE4 carriers compared to non-carriers, and also in patients diagnosed with early AD compared to the cognitively normal. Interestingly, the extent of BBB breakdown was unrelated to Aβ or p-tau pathology. APOE4 is also a risk factor for poor cognitive outcome following TBI, and APOE4 carriers show impaired BBB spontaneous repair following injury compared to non-carriers.^5,109^ However, APOE4 carriers do not appear to be at higher risk of CTE.^110^

Multiple systemic and environmental factors affecting the vasculature have been associated with both increased risk of AD and poor TBI outcome (Table 1). In particular, hypertension, which is associated with AD exacerbation and poor cognitive outcome in TBI (Table 1), has been shown to cause pericyte loss and BBB disruption through upregulation of angiotensin II-dependent proinflammatory pathways.^111,112^ Poor brain perfusion also predisposes to AD and cognitive impairment.^38^ Ischaemia is followed by early and delayed upregulation of tau expression at the site of infarct, with p-tau fragments accumulating in the hippocampal region.^39,113,114^ It also induces amyloidosis through hypoxia-inducible factor 1α (HIF-1α) which upregulates the expression of BACE1, the gene encoding the β-secretase responsible for the beta cleavage of APP.^40,115,116^ HIF-1α plays a central role in the regulation of iron metabolism, providing an intriguing link between ischaemia, oxidative stress and amyloid formation in both TBI and AD. The relationship between these pathologies may be bidirectional, as TBI predisposes to the evolution of cardiovascular disease and further cerebrovascular events.^117,118^ Various mechanisms have been proposed to underlie this link, including neuroendocrine dysregulation with abnormal catecholamine release, increased intracranial pressure and neuroinflammation.^119^ Finally, a host of medications targeting the vascular system appear to help reduce neuroinflammation and improve symptoms in both AD and TBI, further supporting the critical role of the vasculature in their pathogeneses (Table 2).

**Table 2.** Medications originally developed and prescribed for cardiovascular conditions that may have therapeutic benefits in AD and TBI, and their known mechanism of action.

Medication	Original indications of use	Effects in AD and/or TBI	Known mechanism of action
Telmisartan^a^	Hypertension	Improves cognitive abilities and mitigates proteinopathies in AD^120^ Reduces inflammation and oxidative stress in TBI animal models, resulting in partial recovery of motor and cognitive functions^121^	Angiotensin II receptor blocker
Simvastatin	Hypercholesterolemia, prevention of coronary artery disease	Downregulates expression of pro-inflammatory cytokines, reduces microglial activation, promotes neuronal survival, and ameliorates cognitive impairment in AD and TBI^122,123^	Inhibits 3-hydroxy-3-methyglutaryl coenzyme A reductase to lower triglycerides and cholesterol
3K3A-activated protein C (recombinant variant of activated protein C, a natural anticoagulant)	Ischaemic stroke (in development)	Protective against neurovascular damage, reduces perivascular accumulation of fibrinogen and Aβ pathology, inhibits amyloidogenic BACE1 pathway, and mitigates gliosis^124^	Deactivates factors V and VIII of the coagulation cascade
Dabigatran	Anticoagulant commonly used prophylactically in atrial fibrillation patients	Prevents hippocampal fibrin deposition, reduces Aβ pathology, ameliorates neuroinflammation, and rescues cognitive function in AD mouse models^125^	Directly inhibits factor II (thrombin)

The mechanisms underlying their neuroprotective effects, however, are poorly understood.

a Other similar angiotensin antagonists did not show significant neuroprotective effects.

### Plasma protein infiltration and fibrin(ogen) deposition

Plasma protein infiltration is also observed in both AD and TBI, where it is thought to result from increased BBB permeability. This process provides a potential mechanistical link between NVU damage, inflammation mediated by activated microglia, and neurodegeneration.^126^ Indeed, extravascular plasma protein deposits in the cortex and hippocampal region are associated with pericyte degradation in AD patients while albumin influx resulting from loss of tight junctions is frequent in TBI.^96,127^ In particular, there is compelling evidence to suggest that the blood coagulation protein, fibrinogen, plays a key role in this process.

Plasma fibrinogen level positively correlates with total tau and p-tau CSF levels in AD patients, although the mechanisms by which fibrinogen increases tau phosphorylation are yet to be elucidated.^128^ Similarly, although it is not clear whether fibrinogen directly influences Aβ formation, studies have shown that it potentiates the effects of Aβ pathology: Aβ, when in the presence of fibrinogen, specifically binds to the plasma protein to form clots that may exacerbate vascular damage.^129^ Inhibiting this Aβ-fibrinogen interaction reduced vascular Aβ deposition and cognitive impairment in mouse models of AD.^130^ Importantly, fibrinogen has neurotoxic effects independent of Aβ burden in both mouse models and AD patients: induces oxidative stress and microgliosis through binding and activation of microglial CD11b receptors, promoting spine elimination and synaptic loss.^131^ The authors found that cognitive function was rescued by either inhibiting ROS release or deleting CD11b in transgenic mice, which aligns with previously discussed findings whereby BBB damage can result in cognitive dysfunction independently of Aβ or p-tau levels. Corroborating these results, fibrinogen depletion was observed to attenuate microgliosis and AD pathology and to improve cognitive function.^132^ Additionally, AD mouse models lacking 1 allele for plasminogen, the enzyme responsible for fibrinogen proteolysis, show increased Aβ plaque load and cognitive impairment severity.

Fibrinogen deposition in various cortical regions is also associated with neuronal loss and Aβ aggregation in human TBI post-mortem tissues.^133^ Importantly, Muradashvili et al have observed fibrinogen infiltration at the vasculo-astrocyte interface, which activates astrocytes and dependent proinflammatory NF-κB signalling, in turn leading to increased neurodegeneration in TBI mouse models.^134,135^ Infiltration of other plasma proteins such as immunoglobulin G is also detectable but shows no correlation with inflammatory processes or neurodegeneration.

## The Neurovascular Cascade

### Proposed model of neurovascular cascade

**Figure 1.**
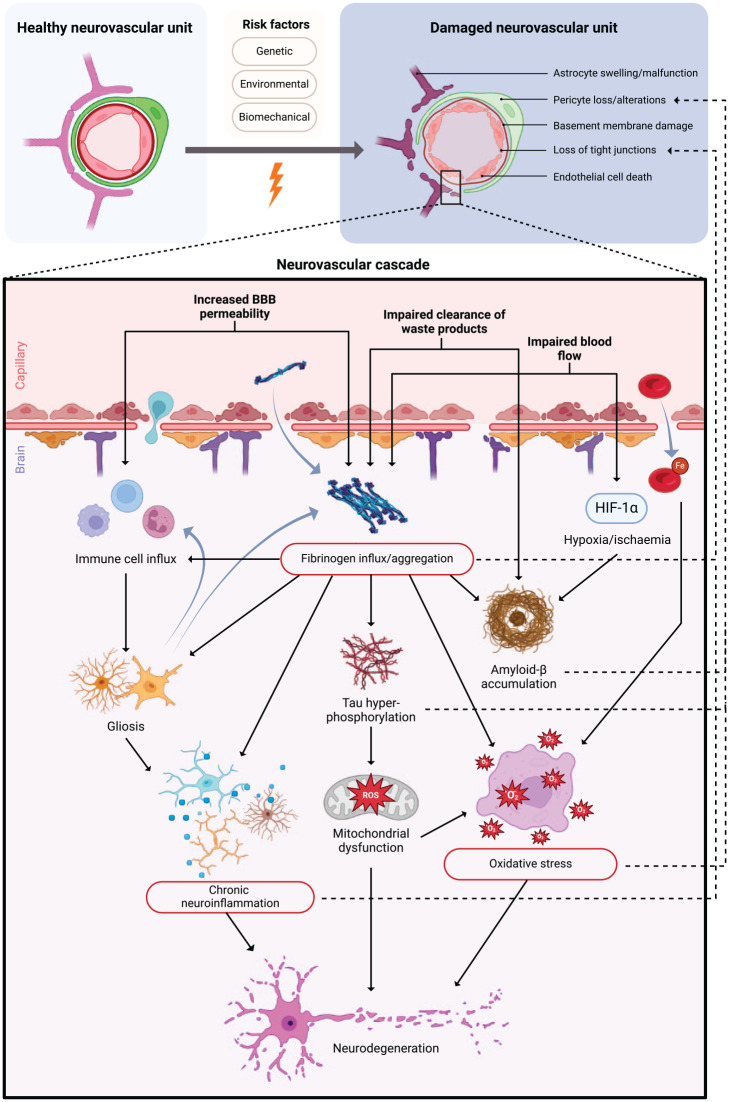
The neurovascular cascade. Initial damage of the NVU leads to a cascade of pathophysiological mechanisms which induce neurodegeneration and further alterations in BBB integrity in a feedforward cycle. Key pathological events which play a role in both inducing neurotoxicity and perpetuating NVU damage are highlighted in red. Created in http://biorender.com/.

This collection of evidence, combining findings from animal models and human post-mortem studies, builds on our existing knowledge of AD and TBI pathophysiology to support the emerging concept of a neurovascular cascade and a critical role for this process in the pathogenesis of both conditions. Based on a synthesis of these results, we propose that high-risk environmental and genetic factors in the case of LOAD, along with mechanical forces in TBI, adversely affect components of the NVU, including tight junctions, basement membranes, endothelial cells, astrocytes and pericytes. These changes may compromise the integrity of the BBB, potentially initiating a cascade of pathophysiological processes centred around the infiltration of fibrinogen into the CNS. The aggregation of fibrinogen could then trigger gliosis, proteinopathies, chronic inflammation and oxidative stress. NVU damage also causes alterations in blood flow and clearance of waste products, which may contribute to the accumulation of fibrinogen, p-tau and Aβ. In addition to their direct neurotoxic effect, these events further exacerbate BBB breakdown to produce a highly damaging feedforward cycle (Figure 1).

A similar combination of neurovascular processes has been observed in numerous neuroinflammatory and neurodegenerative diseases, including multiple sclerosis (MS), Parkinson’s disease, PART and amyotrophic lateral sclerosis (ALS). It is plausible that these conditions, which are also characterised by protein aggregation and neurovascular damage, share common pathophysiological mechanisms by which BBB dysfunction could lead to neuroinflammation and neuronal loss. Thus, the neurovascular hypothesis might serve as a helpful model to address unanswered questions in multiple conditions beyond AD and TBI. A good example of this is the potential role of neurovascular processes in CA1 SNV. This phenomenon has been reported in multiple neurodegenerative diseases including AD, TBI and frontotemporal tauopathies, as well as following cardiovascular events such as transient global ischemia and cardiac arrests.^136,137^ There is growing evidence of a role for tuberous sclerosis complex-1 (TSC1) in protecting hippocampal tissues via inhibition of mTOR signalling.^138,139^ Interestingly, a recent study showed that mouse models lacking the TSC1 gene exhibited aberrant mTOR-dependent vascular endothelial growth factor (VEGF) expression, which led to increased BBB permeability.^140^ Thus, TSC1 dysregulation and/or loss of function in CA1 hippocampal neurons may contribute to their selective vulnerability in both AD and TBI through local loss of BBB integrity. Moreover, AD-mediated pericyte loss begins in the hippocampus, and the same is true of BBB breakdown in normal ageing and mild cognitive impairment.^87,88^ Various pericyte sub-types have been identified, with different morphological characteristics, vessel coverage and vascular territories. Although multiple vascular, inflammatory, and neuronal factors are likely to be involved, it seems at least plausible that SNV of CA1 neurons may partly stem from singularities of a local pericyte subpopulation rendering them particularly vulnerable to damage.^141^ Similarly, heterogeneous astrocyte populations display various structural and functional characteristics in a layer-specific manner across the hippocampus, which might contribute to different profiles of resistance to infarct.^142^

### Implications for treatment

Putting the neurovascular hypothesis into practice, it follows that a number of neurodegenerative and neuroinflammatory pathologies may be alleviated by classes of drugs that act on the vascular system. Indeed, there is a raft of evidence showing that medications originally developed for cardiovascular conditions may be beneficial in both AD and TBI (Table 2). Beyond this evidence, considering the components of the neurovascular cascade, fibrinogen appears to be the most attractive drug target, owing to its pivotal role in our proposed model (Figure 1). With this in mind, it is notable that inhibiting the interaction of fibrinogen with CD11b is sufficient to mitigate neuroinflammatory processes and ameliorate motor function in MS while preserving its essential procoagulant functions.^132,143^ It is also worth mentioning that its derivative, fibrin, appears to play an important part in the cascade: the interaction of fibrin with CD11b-CD18 was shown to be necessary to induce microglia-mediated neurotoxicity in AD and MS mouse models.^143^

### Limitations and outstanding questions

The main limitation of this hypothesis is that the direction of the causal relationship between vascular pathology and proteinopathies remains unknown. It is apparent that Aβ and p-tau accumulation causes neurovascular damage in fAD and, conversely, that BBB opening leads to amyloidosis and tauopathy. Although studies have shown that hippocampal BBB impairment occurs prior to cognitive impairment and neuritic plaque formation in LOAD, the possibility that exposure to soluble oligomeric Aβ is required for increased BBB permeability is worth considering. Significant challenges have impeded the identification of a direct cause-effect relationship; amongst others, the multiplicity of overlapping pathological mechanisms renders it very difficult to control for confounding variables to a satisfactory extent. Furthermore, a reliance on cross-sectional analyses in many studies offers little reassurance regarding the chronological order of different biomarkers.

Regarding temporality, we note in particular the findings of multifactorial data-driven analyses of the spatiotemporal development of pathological mechanisms involved in LOAD throughout disease progression compared to healthy ageing, which found that vascular abnormality was markedly predominant across all time points and brain regions.^144,145^ Cognitive impairment was observed prior to elevated CSF soluble Aβ and p-tau protein levels and progressively increased as other noxious alterations came into play. The strengths of this study include its large sample size, the variety of biomarkers included, and the longitudinal tracking of disease pathology in vivo. However, the heterogeneity of patients with various age-related conditions including cardiovascular pathologies was not controlled for. While these results do not provide definitive evidence of a causal role for vascular pathology in the onset of the disease, they do suggest that neurovascular dysregulation can itself act as an AD-triggering event. Future studies are required to elucidate this point and confirm these findings. Although similar perfusion changes have been observed in the early post-TBI phase and seem to negatively impact patient outcomes in the first 2 years, it is still unclear whether they influence long-term outcomes in chronic TBI.^146,147^

Finally, to build on the neurovascular hypothesis, we suggest three further research questions which ought to be addressed. First, although there is an emerging body of evidence in support of an association between fibrinogen and amyloid pathologies, their mechanistic and causal links remain unclear. Further studies exploring the relationship between both Aβ and p-tau accumulation and fibrinogen infiltration, and its relevance to further escalation of TBI and AD, are required. In view of the role of fibrin in mediating neurotoxic microglial programming, distinguishing between fibrinogen, fibrin and their enzyme cascade is paramount to better understand their respective involvement in the neurovascular cascade. Second, the potential role of other plasma proteins, including those involved in the coagulation cascade, in brain pathology should also be explored: some coagulation factors have been shown to be elevated and associated with neurotoxicity in animal models of various neuroinflammatory and neurodegenerative diseases, and their inhibition, along with that of thrombin, is neuroprotective in mouse models.^124,125,129^ Third, there is growing evidence to suggest that other pathophysiological mechanisms including peripheral insulin resistance and fatty acid metabolism may play a role in inducing and propagating both AD and TBI.^46,144^ Exactly how these mechanisms feed into the neurovascular cascade has yet to be elucidated. Possible mechanistic links include the mTORC1 pathway, which has been shown to be intricately related to insulin resistance and lipid homoeostasis.^148,149^ Its potential role in the pathogenesis of AD and TBI and in mediating SNV of hippocampal neurons also warrants further exploration from the perspective of NVU dysfunction.

## Discussion

The neurovascular link between AD and TBI is increasingly recognised in the literature. Not only is TBI associated with an increased risk of AD, but both pathologies share characteristic histopathological features: amyloidosis, p-tau aggregation, chronic inflammation and oxidative stress all play a critical role in mediating neurodegeneration and cognitive decline in both conditions, albeit following different spatial and temporal evolution patterns. Importantly, emerging evidence suggests that these mechanisms are intimately related to the neurovasculature. NVU damage and BBB breakdown are well-documented in both human and animal models of multiple neuroinflammatory and neurodegenerative diseases and have been shown to trigger a variety of neurotoxic processes. In this review, we propose a common pathway for the initiation and progression of AD and cognitive impairment following TBI: a neurovascular cascade of multifactorial and interlinked pathological mechanisms in which the BBB and plasma proteins including fibrinogen appear to play pivotal roles. This neurovascular hypothesis may also be applicable to other neuroinflammatory and neurodegenerative diseases. We suggest that the pathogenesis of these conditions share common elements which converge upon NVU disruption and BBB dysfunction, while specific combinations of genetic, environmental and biomechanical risk factors would favour certain pathological mechanisms over others, leading to discrete biomarkers and distinctive hallmarks in each condition. This model can serve to guide future studies that seek to elucidate the relationships between these biomarkers, their relative contributions to AD and TBI, and pathways towards effective treatments for both conditions.
